# Knowledge, attitudes and self-confidence with skills required for providing dementia care in physicians at primary healthcare settings in Vietnam

**DOI:** 10.1186/s12913-023-10460-4

**Published:** 2024-01-17

**Authors:** Bich Diep Pham, Bao Giang Kim, Adrian Esterman, Henry Brodaty, Susan Kurrle, Thanh Binh Nguyen, Trong Hung Nguyen, Elizabeth Roughead, Ladson Hinton, Thu Ha Dang, Thi Diem Huong Nguyen, Kham Tran, Maria Crotty, Duc Du, Tuan Anh Nguyen

**Affiliations:** 1 School of Preventive Medicine and Public Health, Hanoi Medical University, Hanoi, Vietnam; 2 UniSA: Allied Health & Human Performance, University of South Australia, Adelaide, SA Australia; 3 Centre for Healthy Brain Ageing (CHeBA), School of Clinical Medicine, University of New South Wales, Sydney, NSW Australia; 4 The University of Sydney, Sydney, NSW Australia; 5 National Geriatric Hospital, Hanoi, Vietnam; 6 University of California, Davis, Sacramento, CA USA; 7 Swinburne University of Technology, Melbourne, VIC Australia; 8 National Ageing Research Institute, Melbourne, VIC Australia; 9 Flinders University, Adelaide, SA Australia; 10 Oxford University Clinical Research Unit, Ho Chi Minh City, Vietnam; 11 Health Strategy and Policy Institute, Ministry of Health of Vietnam, Hanoi, Vietnam; 12 UniSA: Clinical and Health Sciences, University of South Australia, Adelaide, SA Australia

**Keywords:** Knowledge, Attitudes, Self-confidence, Primary health care providers, Vietnam

## Abstract

**Background:**

Dementia is a global public health priority. The World Health Organization adopted a Global Action Plan on Dementia, with dementia awareness a priority. This study examined the knowledge, attitudes, and self-confidence with skills required for providing dementia care among primary health care providers in Vietnam.

**Methods:**

A cross-sectional study was conducted with 405 primary health care providers who worked at commune health stations and district health centers in eight provinces across Vietnam.

**Results:**

The results showed that primary health care providers had poor knowledge and little confidence but more positive attitudes toward dementia care and management.

**Conclusions:**

The results suggest the training needs for building capacity amongst primary health care providers, which will be critical as Vietnam’s population ages.

**Supplementary Information:**

The online version contains supplementary material available at 10.1186/s12913-023-10460-4.

## Background

Dementia is the seventh leading cause of mortality in the world [1]. Fifty million people around the world were estimated to be living with dementia in 2018; a figure that will triple to 152 million by 2050 [2]. Dementia is one of the leading causes of disability and dependence in older adults. It affects not only people with dementia, [3, 4] but also their carers, family, and society [5].

Early diagnosis of dementia enables better preparation for treatment, care, and support for people with dementia and their care [6, 7]. However, published evidence indicates that dementia is underdiagnosed in primary care, with an estimated 61% of undiagnosed dementia cases worldwide [8, 9]. Delayed diagnosis leads to a higher burden of disease and costs to both individuals and the health system [10, 11]. Early diagnosis may be linked to economic benefits, which can translate into long-term cost savings for healthcare systems [12, 13].

The theory of knowledge, attitude and practice shows that any practice is determined by knowledge, and attitude [14]. Many studies have approved the relationship between knowledge, attitude, and practice. Research in China shows that higher levels of knowledge about mild cognitive impairment are likely to have more favorable attitudes about the detection and management of mild cognitive impairment [15, 16]. Additionally, one of the major factors contributing to the underdiagnosis of moderate cognitive impairment in the world is the lack of proper knowledge. Skepticism about the validity of diagnostic criteria for mild cognitive impairment, as well as uncertainty about the progression of it to dementia, may be a barrier for performing screening and diagnosing mild cognitive impairment [16]. A systematic mixed studies review also reported a similar result. The doctor’s belief in the effectiveness of treatment as well as the doctor’s confidence in diagnosing dementia is one of the factors that determine the diagnosis of dementia [17]. Therefore, inadequate knowledge and attitude may lead to the hesistancy in early diagnosis in dementia.

Primary care physicians play an important role in early dementia diagnosis and management [18, 19]. However, studies even in developed countries, show that early diagnosis in the primary care setting is challenging because of limited resources, and lack of knowledge and skills in dementia [20, 21]. Lack of knowledge about dementia has been identified as a key contributor to the global underdiagnosis of dementia [22].

In 2050, two-thirds of people living with dementia will live in low- and middle-income countries [2]. Vietnam, a low-middle-income country, is one of the fastest-aging Asian nations and is predicted to have a dementia burden. The proportion of people aged 65 years and above was 8% in 2020 and will reach 20% by 2050 [23]. Additionally, the number of people aged 80 years and over will triple to 6% of the population by 2050 [24]. In 2015, about 660,000 Vietnamese people were living with dementia, with resultant care costs of US$960 million [25]. The number of people with dementia is predicted to increase to 2.4 million by 2050 [26], thus Vietnam’s health system must be strategically placed to support people with dementia.

In the Vietnamese healthcare system, there are four levels of administrative structure for health care including level I (central), level II (provincial), level III (district), and level IV (commune) [27]. Primary healthcare at the grassroots level, including health care providers at district and commune levels, is a key component of the health care system to ensure that all people can receive the lowest cost and most effective health care close to their communities [28]. However, Vietnam’s primary healthcare system has been under-utilized, and there is an over-reliance on hospitals including provincial or center hospitals. Over-reliance on hospitals might be exacerbated as the number of dementia patients increases in the future [29] if health care workers aren’t skilled at the grassroots level to diagnose and care for people with dementia. Notably, primary health care physicians have provided a diagnosis of dementia for the individual at the first steps in most countries [1]. However, there is a lack of information in Vietnam about the capabilities of primary health care providers in dementia care. This study aimed to understand the current knowledge, attitudes, and self-rated skills in dementia care among primary health care providers in Vietnam. These findings will inform healthcare system planning for an increasing elderly population in Vietnam.

## Methods

### Setting

**Fig. 1:**
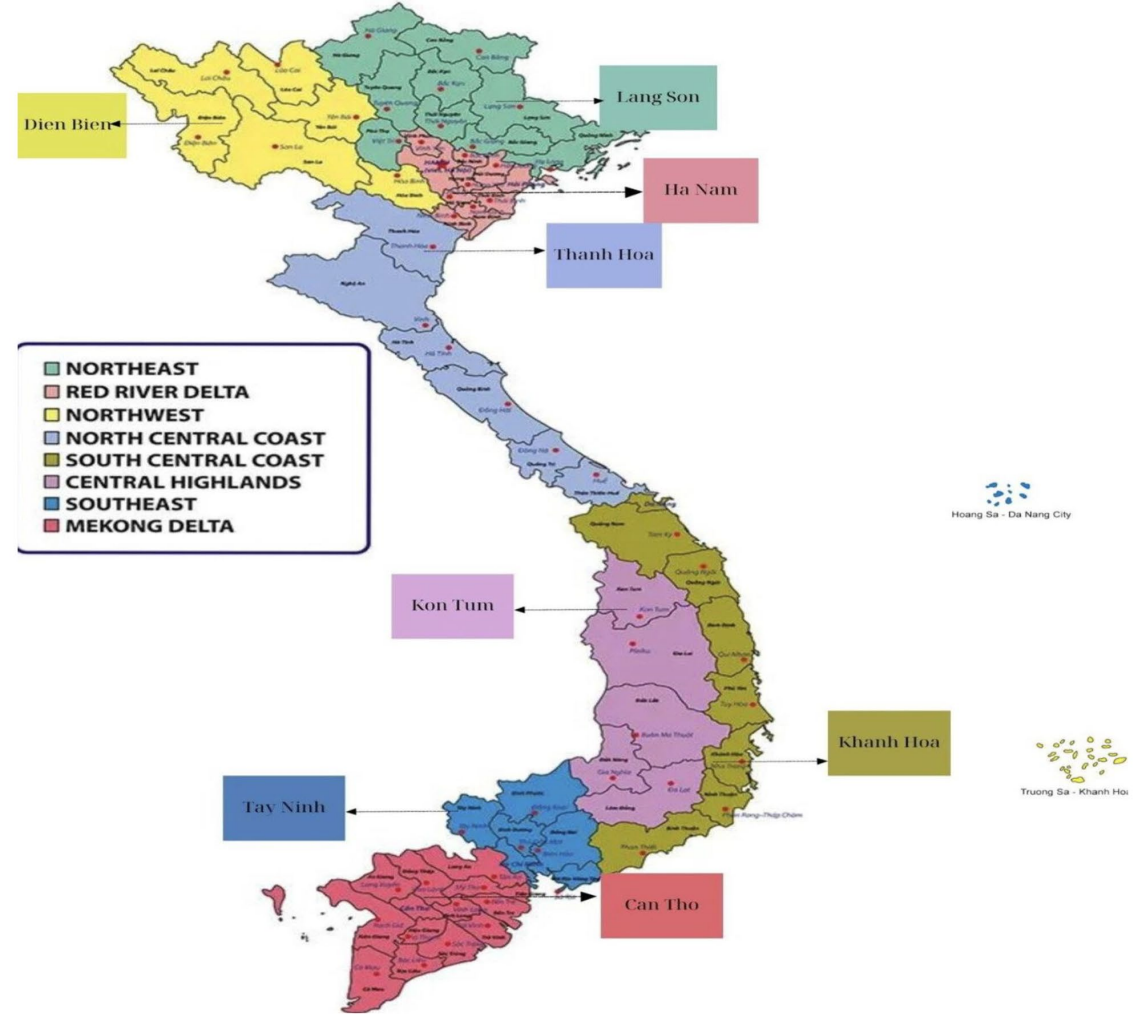
The eight selected provinces in the study

This cross-sectional study was conducted in 2019 in 8 provinces located in the North, the South, the Center and the highland mountainous area, and also representing for the different ecological regions of Vietnam. The eight provinces are shown in the map by different ecological regions (see Fig. 1).

### Sample size and sampling

The World Health Organization sample size calculation formula was used to calculate the sample size. Assuming that the prevalence of correctly answering the questions related to dementia knowledge was 67% among general practitioners [30] (with a precision of ± 0.2 and a 95% confidence level) a sample size of 340 primary health providers was required.

We used a multi-stage stratified sampling approach. Firstly, at the district level, one district with the average economic level was selected in each selected province. Each district has only one district health center responsible for both preventive and treatment tasks. All doctors working in internal medicine and examination departments at the district health center were invited to participate in the study.

Secondly, at the commune level, all health commune stations under the direct management of the district health center were included in the study. The number of health commune stations in each district varied depending on the number of communes per district. Since each health commune station mostly has one doctor who is responsible for the examination and treatment of the commune we invited the doctor to participate in the study. If the commune health station did not have a doctor, we invited the doctor’s assistant or others who had responsibility instead. As a result, there were 405 participants in the study.

### Development of questionnaire

There were two steps in the development of the questionnaire. First, a list of questions was generated, including some adapted from previous surveys on knowledge, attitudes, beliefs, and practice of health professionals on dementia and available dementia knowledge-based tools [31–33]. Additional survey items were developed specifically for this survey, including questions about cultural factors and the primary care provider’s role in dementia prevention and management, traditional medicine practices, and participants’ opinions on dementia training. These items were grouped into themes to produce a draft questionnaire. This was the new questionnaire developed for this study (See the supplementary file 1). The draft English version of the questionnaire was sent to two Australian dementia experts. The inclusion criteria for selecting experts were much experience in dementia including chief investigators of the dementia project; professors with more than 25 years of working experience in dementia; and willingness for this duties. The experts assessed for face and content validity and rated each item as valid, possibly valid, or not valid. The items where it was agreed that they were valid were included. Those items where both experts considered the question invalid were excluded. The research team discussed items with a disagreement or rated possibly valid before a final decision. The panel including two experts from a research team in the dementia area also judged the content validity of the items (i.e. if the content accurately assesses all fundamental aspects of the topic).

The questionnaire was formatted, translated into Vietnamese, and back-translated into English after validity testing of the English version. The Vietnamese-translated version of the questionnaire was sent to a panel of two Vietnamese dementia experts including neuologists working in the national geriatric hospital and dementia research team to rate the face and content validity of each item.

The Vietnamese questionnaire was then piloted among 12 primary care clinicians providers (2 from district health centers and 10 from commune health stations) in Ha Nam province, who were not included in this study. The health care providers were asked to complete the survey twice, one week apart. A member of the research team discussed the clinical sensitivity, questionnaire layout, and question flow with the clinicians who completed the questionnaire. To reduce the recall bias, we did not inform the twelve respondents that we will come back to collect the data in the second times. Additionally, questions in the questionnaire were developed an ease to use and answer. We provided enough time for interviewing each respondent to make sure that he/she has enough time to carefully think and answer the questions.

The reliability of survey items was assessed using the intra-class correlation (ICC) for continuous variables, Gwet’s AC1 for dichotomous and nominal variables, and weighted Gwet’s AC1 for ordinal variables. Any items found to have low reliability were either modified or discarded. The procedure to modify the low-reliability questions followed three steps (1) made phone calls to the respondents and asked them how to understand the questions; (2) used other words to modify the questions (if needed) to make it clearer and prevent misunderstanding; (3) discussed with the experts to make sure that the meaning of questions was the same.

#### Measure

The questionnaire covered six parts.

Part 1: demographic information on gender, age, educational degree, work experience, and professional title.

Part 2: questions that explored the factual knowledge of primary health providers about dementia comprising 13 fixed-response (single choice) questions. The dementia knowledge covered in this paper is the knowledge of primary health care providers on awareness of dementia, epidemiology, risk and protective factors, early symptoms, diagnosis and treatment. Therefore, the knowledge questions pertained to the above topics. Each correct item was scored as one. A total score was calculated by summing the correct scores for each item, yielding a total score ranging from 0 to 13. A higher total score indicated better knowledge.

Part 3: questions that explored primary health providers’ attitudes towards early and pre-dementia diagnosis at the stage of very mild dementia. The attitudes covered in this paper include attitudes toward benefits and risks of early diagnosis, attitudes on treatment options, care people with dementia; and the role of primary health providers in dementia care and management. Attitude questions were chosen and adapted from previous questionnaires [16]. The attitude questions consisted of 16 closed questions measured with a five-point Likert scale (1 = disagree to 5 = agree).

Part 4: Confidence in skills in this study covered the skills in identifying, managing, and distinguishing behavioral and psychological symptoms of dementia; as well as non-pharmacological intervention for diagnosing and treatment dementia consisting of 4 questions with a five-point Likert scale.

Part 5: Practices regarding diagnosis and treatment of dementia at primary healthcare settings in this study including how to diagnose suspect people with dementia, and how to treat and advise people with dementia, which comprised 1 closed question and 3 open-ended questions related to diagnosis and treatment for dementia.

Part 6: Open-ended questions related to further training needs of respondents.

### Data collection

Participation was voluntary and participants were informed about the aims of the study. During the training, ten investigators were trained to ensure that they understood all the questions would follow the same processes, and provide the same explanations to interviewees. The investigators delivered the self-administered questionnaire to the respondents at the regular monthly meeting held at each district health center. If the respondents raised any questions, the investigators answered or explained the questions according to the interview guideline. After each data collection time, new questions raised by respondents were added to the interviewing guideline and unified with all the investigators. The purpose of the study was noted in the questionnaires. Respondents who agreed to participate in the study filled in the questionnaire and returned it to the investigators.

### Analysis

Quantitative data were analyzed using the Stata 15 statistical package. Descriptive statistics including frequency, percentages, means and standard deviations were reported for demographics, knowledge scores, attitudes, and confidence variables. All the correlation between knowledge, attitude and confidence variables were low (≤ 0.3). Qualitative data obtained from open-ended questions on practice in dementia are also listed by content.

## Results

### Characteristics of participants

In total 405 primary health providers from 8 cities (68% at the commune level and 32% at the district level) received and filled in the questionnaires.

Among respondents, 46% were male; 45% were doctors’ assistants and 53% were general or specialised doctors. The health care providers’ number of years of work varied; with 41% having less than 10 years of work experience, 26% having 10 to 19 years of work experience, 24% having 20 to 29 years, and 7% having 30 years and over. Almost all respondents had either a university degree (45%) or a college degree (45%) and nearly one in ten had a postgraduate degree (9%) (Data shown in the Table 1).

**Table 1:** Characteristics of 405 participants in 8 provinces cross Vietnam

Characteristics	District level (n%)	Commune level (n %)	Total (n%)
**Number of respondents by cities**			
Dien Bien/ North West	16 (12.3)	33 (12.0)	49 (12.1)
Kon Tum/ Highland	16 (12.3)	39 (14.2)	55 (13.6)
Can Tho/MK delta	25 (19.2)	23 (8.4)	48 (11.85)
Ha Nam/ Red River Delta	11 (8.5)	36 (13.1)	47 (11.6)
Khanh Hoa/ Southern Central Coastal	15 (11.5)	36 (13.1)	51 (12.6)
Lang Son/ Northern mountainous	7 (5.4)	40 (14.5)	47 (11.6)
Tay Ninh/ Eastern of MK delta	13 (10.0)	35 (12.7)	48 (11.85)
Thanh Hoa/ Northern Central coastal	27 (20.8)	33 (12.0)	60 (14.8)
**Gender**			
Male	51 (39.2)	137 (49.8)	188 (46.4)
Female	79 (60.8)	138 (50.2)	217 (53.6)
**Working position**			
Doctor assistants	4 (3.1)	181 (65.8)	185 (45.7)
General practitioner/specialist doctors	126 (96.9)	92 (33.5)	218 (53.8)
Others	0	2 (0.7)	2 (0.5)
**Education level**			
Post graduate degree	34 (26.1)	4 (1.4)	38 (9.4)
Undergraduate degree	91 (70.0)	92 (33.5)	183 (45.2)
College degree or below	5 (3.9)	179 (65.1)	184 (45.4)
**Work experience**			
Under 10 years	55 (42.6)	111 (40.9)	166 (41.5)
10–19 years	36 (27.9)	69 (25.5)	105 (26.2)
20–29 years	30 (23.3)	69 (25.5)	99 (24.8)
30 years and above	8 (6.2)	22 (8.1)	30 (7.5)
**Age**			
Under 30 years	23 (17.7)	41 (15.0)	64 (15.8)
30–39 years	48 (36.9)	97 (35.5)	145 (36.0)
40–49 years	28 (21.5)	69 (25.3)	97 (24.1)
50 years and above	31 (23.9)	66 (24.2)	97 (24.1)

### Primary health providers’ dementia knowledge

Table 2 shows the primary health providers’ responses to the dementia knowledge questions. The total mean score of the primary health providers’ dementia knowledge was 6.9 (SD = 2.1) (accounting for 53% of correct answers), out of a total of 13 questions, suggesting knowledge gaps. Results showed areas of concern regarding knowledge of “post-mortem procedures can confirm a diagnosis of dementia” and “When a person develops a sudden onset of confusion, disorientation, and inability to sustain attention, this presentation is most consistent with the diagnosis of Delirium”.

**Table 2:** Primary health providers’ dementia knowledge

Items	Number of participants at the commune level answered correctly (n;%) (n = 275)	Number of participants at the district level answered correctly (n;%) (n = 130)	Total (n; %) (n = 405)	*P* value
**Definition**				
Dementia is a decline in mental ability severe enough to interfere with everyday life	173 (62.9)	79 (60.8)	252 (62.2)	> 0.05
**Early symptom and Diagnosis**				
Early symptom of Alzheimer’s disease is impaired memory.	197 (71.6)	108 (83.1)	305 (75.3)	< 0.05
Post-mortem procedures can confirm a diagnosis of dementia.	2 (0.7)	0 (0)	2 (0.5)	> 0.05
Delirium can be caused by Infection, Dehydration, Medication.	223 (81.1)	111 (85.4)	334 (82.5)	> 0.05
When a person develops a sudden onset of confusion, disorientation, and inability to sustain attention, this presentation is most consistent with the diagnosis of Delirium	26 (9.5)	16 (12.3)	42 (10.4)	> 0.05
It is important to rule out and treat reversible diseases	102 (37.1)	51 (39.2)	153 (37.8)	> 0.05
**Treatment**				
Not all types of dementia are progressive and can be cured	192 (69.8)	83 (63.8)	275 (67.9)	> 0.05
Anti-dementia drugs can temporarily halt or slow the progression of symptoms in some patients	185 (67.3)	97 (74.6)	282(69.6)	> 0.05
If someone with dementia is also depressed, anti-depressant drugs can sometimes work	172 (62.5)	74 (56.9)	246 (60.7)	> 0.05
**Risk and protective factors**				
Depression, some medications and stress can cause memory loss	227 (82.5)	115 (88.5)	342 (84.4)	> 0.05
A good balance diet helps brain functioning in healthy adults	87 (31.6)	39 (30.0)	126 (31.1)	> 0.05
Drinking a lot of alcohol decreases brain functioning in healthy adults.	131 (47.6)	76 (58.5)	207 (51.1)	< 0.05
**Epidemiology**				
5-10% of people age over 65 are likely to have dementia.	149 (54.2)	72 (55.4)	221 (54.6)	> 0.05
**Total score of knowldege (Mean; SD)- Median(Min-Max)**	(6.8; 2.1)- 7.0 (0; 11)	(7.1; 1.9)- 7.0 (2; 11)	(6.9; 2.1) – 7.0 (0; 11)	> 0.05

### Primary health care providers’ attitudes to dementia care

**Fig. 2:**
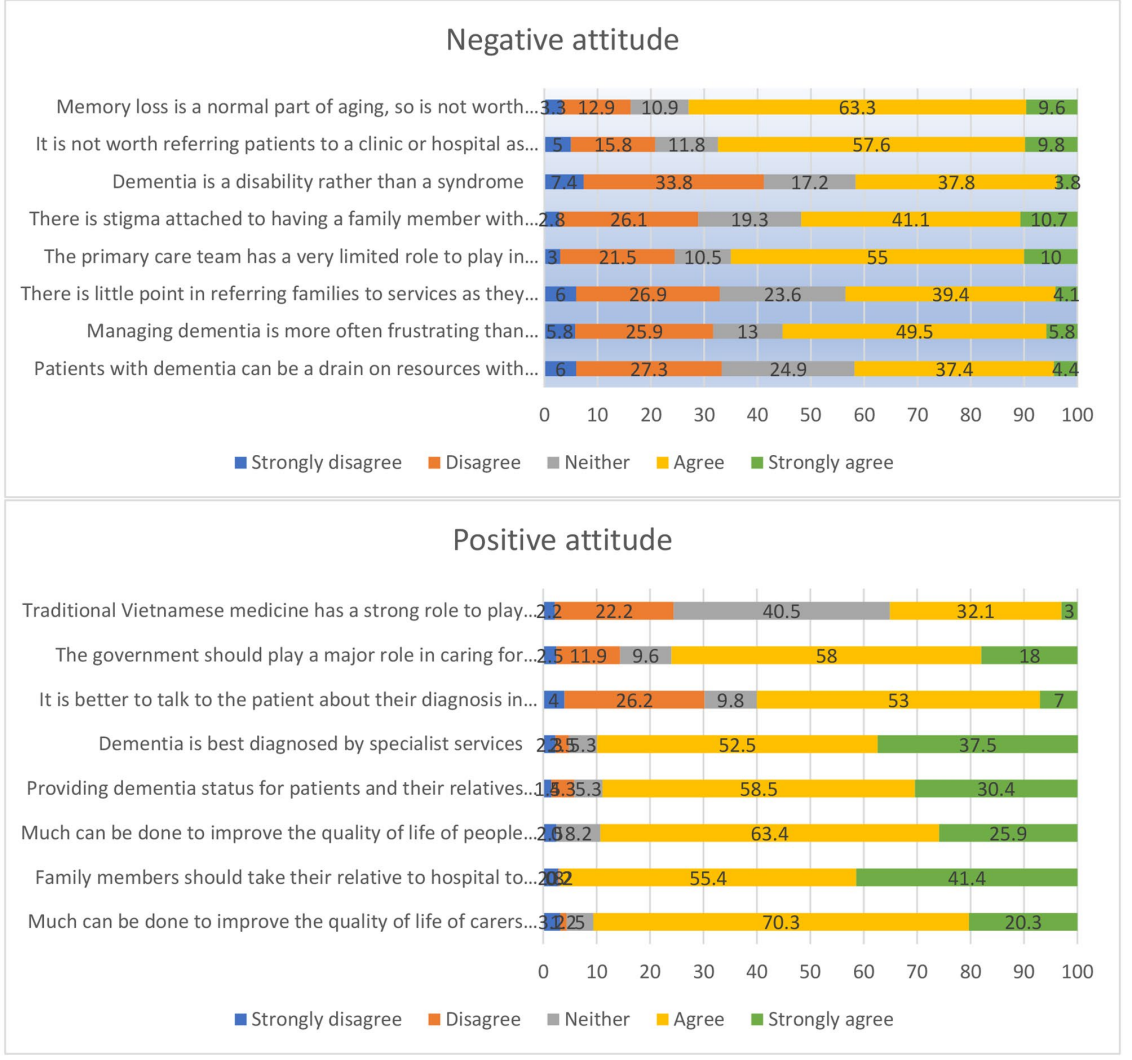
Primary health providers’ positive and negative attitudes to dementia care (n = 405)

Figure 2 shows the health care providers’ agreement rate with the questionnaire items related to attitude. The results show attitudes were much more positive towards the following items: “Family members should take their relative to the hospital to know their relative’s dementia as soon as possible”; “Much can be done to improve the quality of life of carers of people with dementia and people with dementia”; “providing dementia status for patients and their relatives is usually more helpful than harmful”; “dementia is best diagnosed by specialist services”; while attitudes were much negative towards the following items: “memory loss is a normal part of aging, so is not worth treating”; “It is not worth referring patients to a clinic or hospital as travel is too difficult or expensive”; “the primary care team has a very limited role to play in the management and care of people with dementia”.

### Primary health providers’ self-confidence and belief about behavior and psychological symptom of dementia

Less than half the participants reported they felt confident in dementia care. Few respondents felt confident in identifying, diagnosing, and managing behavior and psychological symptoms of dementia. The highest confidence was reported for the item “believe that non-pharmacological interventions have a major role in managing behavioral and psychological symptoms of dementia” and the lowest percentage was for the item “I have sufficient skills to manage behavioral and psychological symptoms of dementia”. Detailed results are displayed in Table 3.

**Table 3:** Primary health providers’ self-confidence on their dementia care skills

Items	Number of physicians strongly disagree (n, %)	Number of physicians disagree (n, %)	Neither (n, %)	Number of physicians agree (n, %)	Number of physicians strongly agree (n, %)	Mean ± SD
I have sufficient skills to identify behavioural and psychological symptoms of dementia	13 (3.2)	78 (19.3)	182 (44.9)	127 (31.4)	5 (1.2)	3.1 (0.8)
I have sufficient skills to manage behavioural and psychological symptoms of dementia	10 (2.5)	116 (28.6)	186 (45.9)	91 (22.5)	2 (0.5)	2.9 (0.8)
I have sufficient skills to distinguish between behavioural and psychological symptoms of dementia and other behavioural disturbance not related to dementia	10 (2.5)	94 (23.2)	180 (44.4)	119 (29.4)	2 (0.5)	3.0 (0.8)
I believe that non-pharmacological interventions have a major role in the management of behavioural and psychological symptoms of dementia	10 (2.5)	83 (20.5)	120 (29.6)	179 (44.2)	13 (3.2)	3.3 (0.9)

### Primary health providers’ practices on dementia diagnosis and treatment

Table 4 shows the answers to the questions about primary health providers’ practices on dementia diagnosis and treatment. Nearly 90% of participants reported that they would refer dementia patients to someone else for diagnosis and treatment. Among 405 respondents, only 10% answered the open-ended questions related to diagnosis, treatment, and instruction for dementia.

**Table 4:** Primary health providers’ practices on dementia diagnosis and treatment (n = 405)

Question	Answers	Number	%
If you think that one of your patients might have dementia, do you:	Diagnose and manage their care yourself	44	10.9
	Refer them to someone else for diagnosis and treatment	361	89.1
How would you make a diagnosis of dementia?	44 responses: clinical assessment, Mini mental state examination (MMSE), age group, symptoms of memory lost, sickness history, tests on memory and attention, observation, mental and psychological tests		
What treatment	44 responses around: - Traditional medicines - Non-pharmacological treatment - General treatment - Focusing on behaviour - Changing social life context - Rehabilitation - Physical exercises - Attending social clubs - Stress/depression treatment Special medicines		
What advices/instructions	44 responses around: - Changing eating habits to more wheats, vegetables of Vitamin B group - Changing attitudes on caring - Social supports, encouragement - Dementia information and caring information - Social connectedness and encouragement of social interaction/communication - Do not panic - Be positive - Paying more attention Reducing triggers		

More than 90% of respondents reported that they needed to have further training in dementia prevention, diagnosis, treatment, and care.

**Fig. 3:**
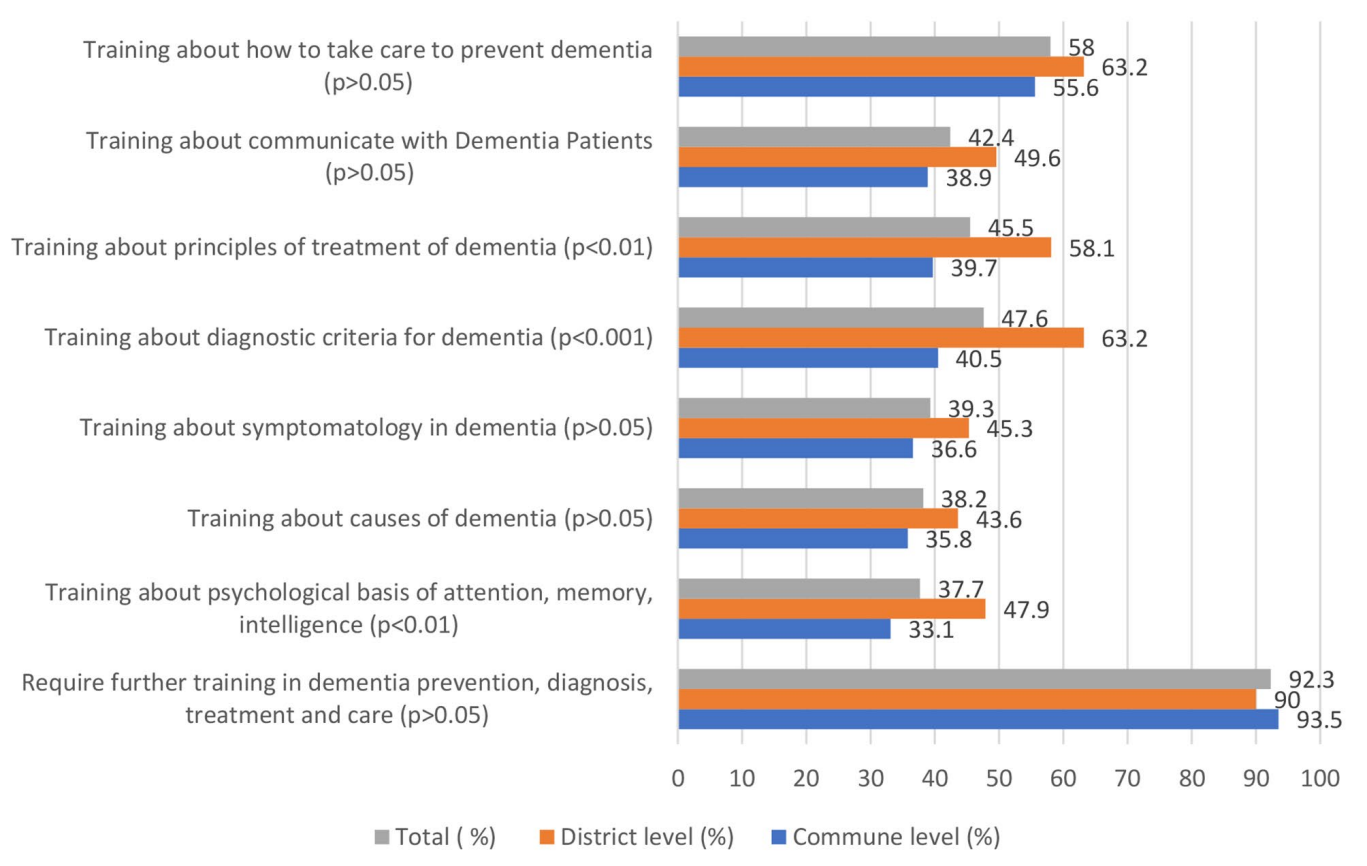
Further training needs in dementia among primary health providers (n = 405)

Figure 3 reports the training needs in dementia from the point of view of primary health providers. More respondents at the district level reported training needs than respondents at the commune level. Interestingly, there were significant differences between commune and district levels regarding need for training in “psychological basis of attention, memory, intelligence”; “epidemiology of dementia”, “diagnostic criteria for dementia”, and “principles of treatment of dementia”.

## Discussion

Our study examines the knowledge, attitudes, and self-confidence with skills in the diagnosis and management of dementia among primary health care providers in Vietnam. It is of fundamental importance to understand the community health professionals’ perspectives who play an important role in responding to the increasing number of people living with dementia in Vietnam. When understating the capacity of community health professionals, the government can provide appropriate educational interventions, and policies can be designed and implemented to develop a competent dementia workforce in the community setting.

This study suggests gaps in dementia knowledge among primary health care providers in Vietnam. The mean knowledge score was 6.9 (about 53% of correct answers), and nobody obtained 100% correct answers. We could not directly compare the knowledge between respondents in our study and other studies. However a study in China revealed that the mean knowledge score was 20 out of 30 (66% correct answers) and in the US the score was 27 out of 30 [32], (90% correct answers). The poor knowledge in our study might be explained by the fact that dementia diagnosis and treatment is only taught one lesson in the training curriculum of undergraduate medical students. Additionally, although primary health care providers in Vietnam are required to attend continuing professional development programs to gain 48 continuous training hours per two years, [34] dementia is not a compulsory topic for continuing professional development. Furthermore, most annual training programs from the Direction of Healthcare Activities (Chi dao tuyen) for staff working at the primary health level rarely include dementia diagnosis, treatment, and management. Notably, our study shows no difference in dementia knowledge between providers at commune and district levels except for two statements “impaired memory is an early symptom of Alzheimer’s disease” and “drinking a lot of alcohol decreases brain functioning in healthy adults”. This result might be explained by the fact that all primary health care providers are less exposed to people with dementia therefore there is no difference in knowledge between the two levels of the primary healthcare system. In Vietnam, people with dementia are diagnosed by neurologists at special national or provincial hospitals. However, there are only 27 hospitals and clinics provide dementia diagnosis and treatment, which are mostly located in the two biggest cities including Hanoi and Hochiminh [34]. Therefore, primary health care providers should be trained to recognize high-risk people with dementia and then refer them to a higher level for diagnosis.

The study reveals that primary health care providers have a positive attitude to dementia care and management. A high percentage of primary health care providers generally agreed and strongly agreed with more positive attitudes except for the statement “Traditional Vietnamese medicine has a strong role to play in dementia”. The findings reveal that most primary health care providers recognized the importance of dementia diagnosis, management, and care. Similar findings have been reported in other countries including China [35] and the US [32]. Our study shows that 65% of primary health care providers recognized or understood their role in dementia management. There was a much smaller proportion of primary health care providers (20–30%) who believed that “It is not worth referring patients to a clinic or hospital as travel is too difficult or expensive” and “Managing dementia is more often frustrating than rewarding”. These beliefs suggest that a higher percentage of primary health care providers had a generally positive attitude toward dementia care and management. The positive finding suggested that primary health care providers might consider dementia care as a part of their duties, which is similar to other chronic diseases they are managing. In Vietnam, there is a national chronic disease prevention program including hypertension and diabetes that comprises a care approach to manage and screen high-risk people at the commune health centers and refer them to higher levels for confirmation diagnosis [36]. This model has been working well at the grassroots level and has helped to decrease the workload for the higher levels. Therefore, the existing model in hypertension and diabetes management in primary healthcare settings could apply to dementia care and management.

Notably, less than 50% of respondents felt confident in diagnosing and managing dementia. The confidence in our study is lower than that in other studies in China (self-confidence in dementia care skills was 53.93 out of 75) [16] and Malaysia (mean confidence score = 2.96 out of 5) [37]. Our questions in the confidence scale were “ability to identify/manage behavioral and psychological symptoms of dementia”; and “distinguish between behavioral and psychological symptoms of dementia and other behavioural disturbance not related to dementia”. The explanation for the low confidence might be the lack of knowledge and also the lack of screening tools that can be used at the primary healthcare center. Research showed that screening tools can be effectively used at the primary healthcare level and then referring patients to the confirmatory diagnosis at the special hospital level [38].

In our study, less than half of respondents agreed with the role of non-pharmacological interventions in dementia treatment and management, while this number in Israel was nearly double [39] (96% of physicians in the study were aware of the importance of non-pharmacological interventions). The difference between the findings in the two countries might explain that in Vietnam, confirmation of diagnosis and treatment for dementia is mostly provided by specialists at national or provincial hospitals but not in primary health care centers. Therefore primary health providers are not familiar with the dementia treatment including non-pharmacological interventions.

Our study results also show that almost all primary health care providers reported that they needed to have further training in dementia prevention, diagnosis, treatment, and care. Many researchers reported the effectiveness of training can help to improve knowledge of dementia [15, 40]. A study in Malaysia approved that an increase in the knowledge as well as attitude score can increase the confidence score [37]. Since Vietnam will become an aged country by 2050 when people over the age of 65 will predictably account for about 20% of the population, training primary health care providers to prepare dementia workforce in the future is much necessary. Facing this problem, the National Plan for Prevention and Control of Non-Communicable Diseases (NCD) 2022–2025 was approved in 2022, which mentions Mental Health Disorders as one part of the Plan. It is the first time mental health disorder was mentioned in the national Plan, showing government attention to dementia area [41]. Therefore, to obtain the objective of the National Plan, the Vietnamese government should follow the World Health Organization (WHO) suggestion that effective intervention in dementia areas should involve primary care settings [42]. To do that, the early diagnosis and management guidelines for dementia in primary care should be issued. Additionally, screening tools for early diagnosis of dementia should be introduced and trained for the primary health care providers. Furthermore, annual training programs from the Direction of Healthcare Activities (Chi dao tuyen) should plan the training focus on screening and management of dementia for primary health care providers. Dementia services in Vietnam are concentrated in the hospital system, which is mostly located in the two biggest cities. Other cities of Vietnam have provincial hospitals with geriatrics departments, however, there are no specialists who can provide a diagnosis for people with dementia so patients are referred to higher specialist hospitals [34]. Therefore, it is necessary to think about dementia care and management models that have been approved and effective in Western countries [43]. Integrated and collaborative care should involve primary healthcare centers, social services, and hospitals that help to provide comprehensive care for dementia patients.

Further study should focus on implementation research for a pilot integrated and collaborated dementia care in Vietnam. Based on the pilot result, might suggest an effective model for the Vietnamese context.

## Conclusion

Primary health care providers in Vietnam’s healthcare system play an important role, however, they have poor knowledge and confidence but more positive attitudes toward dementia care and management. The government could consider developing a national program training for primary health care providers and increase awareness of the population about dementia care and management.

In this way, Vietnam can prepare for the future of a rapidly aging population and improve the quality of life for people with dementia and their family caregivers, which leads to reduce the burden on healthcare and social care systems.

### Electronic supplementary material

Below is the link to the electronic supplementary material.

Supplementary Material 1

## Data Availability

The datasets used and/or analysed during the current study are available from the corresponding author on reasonable request.
