# Supplementary material for: Knowledge, attitudes and self-confidence with skills required for providing dementia care in physicians at primary healthcare settings in Vietnam
Source: BMC Health Serv Res. 2024 Jan 17;24:86. doi: 10.1186/s12913-023-10460-4 (PMC10792887; doi:10.1186/s12913-023-10460-4)
Supplement: Supplementary file 1 — Supplementary Material 1 [file 12913_2023_10460_MOESM1_ESM.doc]

| 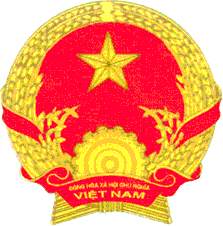 | 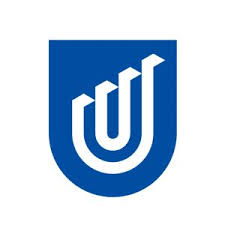 |
| --- | --- |
| **Ministry of Health** | **University of South Australia** |

**PRIMARY HEALTH CARE CLINICIANS SURVEY ON DEMENTIA**

**Introduction**

Hi, I am ……….. from ………. working for a research project of Ministry of Health of Vietnam and the University of South Australia, Australia.

Thank you for agreeing to take part in this survey, which asks about your knowledge, attitude and health practices towards people with dementia. Your answers to these questions will help our country develop a national policy on dementia and improve services to people with dementia and their carers.

We will ask you some questions for less than 30 minutes. Please note that your answers to these questions will remain anonymous, as only summarised data will be reported. Some of the questions are open-ended, that is, they ask for your response in terms of words. We do encourage you to respond to them as they often provide more insight than just ticking boxes.

If at any time after this interview you have any questions or would like to speak to someone involved in this research, please feel free to contact the School of Pharmacy and Medical Sciences, the University of South Australia or the Ministry of Health of Vietnam, 138 Giang Vo, Ba Dinh, Ha Noi. Tel. (04)

Do you have any questions?

Do you agree to participate?

No: THANKS and FINISH the interview

Yes: CONTINUE

Interviewer’s name and Signature: ....................................Date: ......................

| **FOR INTERVIEWER** | | | |
| --- | --- | --- | --- |
| I0 | Name of hospital/clinic |  | |
| I1 | Commune |  | |
| I2 | District |  | |
| I3 | Province |  | |
| I4 | Interview date | Date……….Month……….Year……… | |
| I5 | Interviewer’s name |  | Interview’s code: |

**Please read out:**

| **What is dementia?**  Just to remind you.  Dementia describes a collection of symptoms that are caused by an illness of the brain. It is not just one disease. Dementia affects thinking, behaviour and the ability to perform everyday tasks. The way the brain works is affected enough to interfere with the person’s normal social or working life. The key feature of dementia is the inability to carry out everyday activities as a consequence of diminished ability to think properly. |
| --- |

**A. DEMOGRAPHICS**

| A1. | Gender (*observe*) | Male  Female | 1  2 |
| --- | --- | --- | --- |
| A2. | How old are you? | 18-24  25-34  35-44  45-54  55-64  65-74  75+ | 1  2  3  4  5  6  7 |
| A3. | What is your highest education? | School only  Certificate or diploma  Undergraduate degree  Postgraduate degree  Other (specify)……………….… | 1  2  3  4  8 |
| A4. | What is your occupation? | Community health worker  Nurse  Primary care physician  Social worker  Psychologist  Psychiatrist  Geriatrician  Neurologist  Other (specify)……………….… | 1  2  3  4  5  6  7  8  9 |
| A5. | What is your marital status? | Single – never married  Married/Remarried  Separated  Divorced/Widowed | 1  2  3  4 |
| A6. | Where do you usually work?  *.* | Village  Community Health Centre  District Health Clinic  Hospital  General practice  Other (specify)…………………. | 1  2  3  4  5  8 |
| A7. | How many years have you worked since you were qualified? |  | |
| A8. | In the last 12 months, approximately how many people with dementia have you seen for advice or treatment? |  | |
| A9. | What are the local names and terms for dementia? | | |
| A10. | What does your community think about people with dementia? | | |

**B. KNOWLEDGE OF DEMENTIA**

The next section asks you about your knowledge of dementia. Do not get worried if you give the incorrect answer or do not know the answer.

| B1. | What is dementia? *.* | | | |
| --- | --- | --- | --- | --- |
|  | A condition involving memory loss that is separate from Alzheimer’s Disease  A decline in mental ability severe enough to interfere with everyday life Another word for Alzheimer’s  Not sure | 1  2  3  4 | |  |
| B2. | Which of the following is an early symptom of Alzheimer’s disease? *.* | | | |
|  | Deterioration in communication  Impaired judgement  Impaired memory  Loss of mobility  Not sure | 1  2  3  4  5 | |  |
| B3. | Is the following statement true?  All types of dementia are progressive and cannot be cured | | | |
|  | True  False  Not sure | 1  2  3 | |  |
| B4. | Which of these can cause memory loss? | | | |
|  | Depression  Some medications  Stress  All of them  Not sure | 1  2  3  4  5 | |  |
| B5. | Which of these helps brain functioning in healthy adults? | | | |
|  | Doing puzzles  Exercise  A good balanced diet  Not sure | 1  2  3  4 | |  |
| B6. | Which of these decreases brain functioning in healthy adults? | | | |
|  | Smoking  Drinking coffee  Lack of socializing with other people  Drinking a lot of alcohol  Not sure | 1  2  3  4  5 | |  |
| B7. | What proportion of people age over 65 are likely to have dementia? | | | |
|  | 2%  5%-10%  Not sure | 1  2  3 | |  |
| B8. | If you think a person has dementia, they should be evaluated as soon as possible because:  *.* | | | |
|  | Prompt treatment can delay progression  It is important to rule out and treat reversible diseases  They should be hospitalized as soon as possible  Not sure | 1  2  3  4 |  | |
| B9. | Delirium can be caused by: | | | |
|  | Infection  Dehydration  Medication  All of the above  Not sure | 1  2  3  4  5 |  | |
| B10. | Which of these procedures can confirm a diagnosis of dementia? |  |  | |
|  | Mini-mental State Exam  CAT scan of the brain  Post-mortem  Not sure | 1  2  3  4 |  | |
| B11. | When a person develops a sudden onset of confusion, disorientation, and inability to sustain attention, this presentation is most consistent with the diagnosis of: | | | |
|  | Alzheimer’s Disease  Major depression  Delirium  Not sure | 1  2  3  4 |  | |
| B12. | Anti-dementia drugs can: | | | |
|  | Permanently halt the disease  Temporarily halt the disease in all patients  Temporarily halt or slow the progression of symptoms in some patients  Not sure | 1  2  3  4 |  | |
| B13. | IF someone with dementia is also depressed: | | | |
|  | Anti-depressant drugs can sometimes work  Sadness is part of the disease and not worth treating  Antidepressant drugs should not be given  Not sure | 1  2  3  4 |  | |

**C. ATTITUDE TOWARD DEMENTIA**

Now, I will read some statements about dementia: please say whether you strongly disagree, disagree, are unsure, agree or strongly agree with each statement.

| C1. | Much can be done to improve the quality of life of carers of people with dementia. | | | | | |
| --- | --- | --- | --- | --- | --- | --- |
|  | Strongly disagree | Disagree | Unsure | Agree | Strongly agree |  |
|  | **1** | **2** | **3** | **4** | **5** |  |
| C2. | Family members should bring their relatives to the examination to be diagnosed about their relative’s dementia as soon as possible | | | | | |
|  | **1** | **2** | **3** | **4** | **5** |  |
| C3. | Much can be done to improve the quality of life of people with dementia | | | | |  |
|  | **1** | **2** | **3** | **4** | **5** |  |
| C4. | Providing diagnosis is usually more helpful than harmful | | | | |  |
|  | **1** | **2** | **3** | **4** | **5** |  |
| C5. | Dementia is best diagnosed by specialist services | | | | |  |
|  | **1** | **2** | **3** | **4** | **5** |  |
| C6. | Patients with dementia can be a drain on resources with little positive outcome | | | | | |
|  | **1** | **2** | **3** | **4** | **5** |  |
| C7. | It is better to talk to the patient about their diagnosis in euphemistic terms | | | | | |
|  | **1** | 2 | **3** | **4** | **5** |  |
| C8. | Managing dementia is more often frustrating than rewarding | | | | | |
|  | **1** | **2** | **3** | **4** | **5** |  |
| C9. | There is little point in referring families to services as they do not want to use them | | | | | |
|  | **1** | **2** | **3** | **4** | **5** |  |
| C10. | The primary care team has a very limited role to play in the care of people with dementia | | | | | |
|  | **1** | **2** | **3** | **4** | **5** |  |
| C11. | There is stigma attached to having a family member with dementia | | | | | |
|  | **1** | **2** | **3** | **4** | **5** |  |
| C12. | Traditional Vietnamese medicine has a strong role to play in dementia | | | | | |
|  | **1** | **2** | **3** | **4** | **5** |  |
| C13. | Dementia is a disability rather than a syndrome | | | | | |
|  | **1** | **2** | **3** | **4** | **5** |  |
| C14. | The government should play a major role in caring for people with dementia | | | | | |
|  | **1** | **2** | **3** | **4** | **5** |  |
| C15. | It is not worth referring patients to a clinic or hospital as travel is too difficult or expensive | | | | | |
|  | **1** | **2** | **3** | **4** | **5** |  |
| C16. | Memory loss is a normal part of aging, so is not worth treating | | | | | |
|  | **1** | **2** | **3** | **4** | **5** |  |

**D. PRACTICE TOWARD DEMENTIA**

| D1. | If you think that one of your patients might have dementia, do you:  . | | | Diagnose and manage their care yourself  Refer them to someone else for diagnosis and treatment | | | 1  2 | **🡪 D2**  **🡪 D5** |
| --- | --- | --- | --- | --- | --- | --- | --- | --- |
| D2. | How would you make a diagnosis of dementia? | | | | | | | |
|  | | | | | | | |
| D3. | What treatment if any would you recommend? | | | | | | | |
|  | | | | | | | |
| D4. | What advice/instructions would you give to the patient’s carer? | | | | | | | |
|  | | | | | | | |
| D5. | I have sufficient skills to identify behavioural and psychological symptoms of dementia | | | | | | | |
|  | Strongly disagree | Disagree | Unsure | | Agree | Strongly agree | | |
|  | 1 | 2 | 3 | | 4 | 5 | | |
| D6. | I have sufficient skills to manage behavioural and psychological symptoms of dementia | | | | | | | |
|  | 1 | 2 | 3 | | 4 | 5 | | |
| D7. | I have sufficient skills to distinguish between behavioural and psychological symptoms of dementia and other behavioural disturbance not related to dementia | | | | | | | |
|  | 1 | 2 | 3 | | 4 | 5 | | |
| D8. | I believe that non-pharmacological interventions have a major role in the management of behavioural and psychological symptoms of dementia | | | | | | | |
|  | 1 | 2 | 3 | | 4 | 5 | | |

**E. GENERAL QUESTIONS**

| E1. | Do you require further training in dementia prevention, diagnosis, treatment and care? | Yes  No | 1  2 | **🡪 E2**  **🡪 E3** |
| --- | --- | --- | --- | --- |
| E2. | If Yes, what training would you like, and where should it take place? | | | |
|  | Psychological basis of attention, memory, intelligence  Epidemiology of dementia  Causes of dementia  Symptomology in dementia  Diagnostic criteria for dementia  Principles of dementia treatment  How to communicate with dementia patients  How to care to prevent dementia  Other (specify)…………………………….. | | **1**  **2**  **3**  **4**  **5**  **6**  **7**  **8**  **9** | |

***Thank you for completing the survey!***
